# Human urinary cells for functional wound healing with sweat gland restoration

**DOI:** 10.1186/s40779-023-00492-6

**Published:** 2023-11-28

**Authors:** Shuai-Fei Ji, Lai-Xian Zhou, Ying-Ying Li, Jiang-Bing Xiang, Hua-Ting Chen, Yi-Qiong Liu, Xiao-Bing Fu, Xiao-Yan Sun

**Affiliations:** Research Center for Tissue Repair and Regeneration Affiliated to the Medical Innovation Research Department and 4th Medical Center, Chinese PLA General Hospital and PLA Medical College/PLA Key Laboratory of Tissue Repair and Regenerative Medicine and Beijing Key Research Laboratory of Skin Injury, Repair and Regeneration/Research Unit of Trauma Care, Tissue Repair and Regeneration, Chinese Academy of Medical Sciences, Beijing, 100048 China

**Keywords:** Urinary cell, Urinary stem cell, Wound healing, Sweat gland restoration

Dear Editor,

The large defects of the skin caused by burns or trauma can result in disruption of the structure and function of the skin, as well as irreversible loss of skin appendages, such as sweat glands (SG). As the important modulator of temperature homeostasis, SG damage triggers heat intolerance and thermoregulatory dysfunction, which poses a considerable threat to health and quality of life [1–3]. The promotion of wound healing with SG restoration remains a challenging issue. It has been reported that there are urinary epithelial cells (UECs) in human urine samples, and they may most likely originate from renal proximal tubules [4]. UECs are easy to obtain from urine samples at any age, sex, or ethnic origin except for renal failure, with a cost-effective, non-invasive and simple isolation method [4]. More importantly, the extensive source of clinical urine sample makes it easy for urinary cell autogenous transplantation and individualized treatment. Therefore, it is of great significance to convert UECs into urinary epithelial stem cells (UESCs) with repair ability. In this context, we design a strategy for the pharmacological conversion of UECs into UESCs, and the repair ability of UESCs in functional skin wound healing is explored.

As shown in Fig. 1a, UECs were isolated from 10 healthy male adult donors, and then cultured in renal epithelial cell growth medium with a chemical cocktail consisting of SB431542 (10 μmol/l), Forskolin (10 μmol/l), TTNPB (1 μmol/l), bone morphogenetic protein 4 (20 ng/ml), keratinocyte growth factor (20 ng/ml), epidermal growth factor (20 ng/ml)-inducing medium (SFTBKE) (Additional file 1: Table S1). The initial UEC viability is 95–98% (Additional file 1: Fig. S1a). Briefly, UECs were cultured in the inducing medium for 7 d, and then, the SFTBKE-induced epithelial stem cell-like morphology emerged (Fig. 1b). Furthermore, these induced cells could be passaged more than 10 times with an epithelial morphology, while the third‐generation UECs nearly lost the epithelial morphology and proliferative capacity (Fig. 1c). Besides, the UESCs have stable expansion ability when continuous in vitro passage, but UEC not (Additional file 1: Fig. S1b). RNA expression profiling by quantitative real-time polymerase chain reaction revealed a significant increase in levels of stem cells-associated markers including epithelial cell adhesion molecule, SRY-box transcription factor 9, cytokeratin (CK) 19, leucinerich repeat containing G protein-coupled receptor 6 (LGR6) and tumor protein 63 in SFTBKE-induced cells (Fig. 1d) [5–7]. Colony formation assay results showed that such induced cells were more efficient in forming larger and more colonies compared with UECs, demonstrating the effective impact of chemical cocktails on inducing UECs stemness and proliferation (Fig. 1e, f). The stem cell marker, LGR6, was also verified by immunostaining (Fig. 1g). Taken together, these data indicated that UECs have been induced into UESCs after exposure to SFTBKE.

**Fig. 1:**
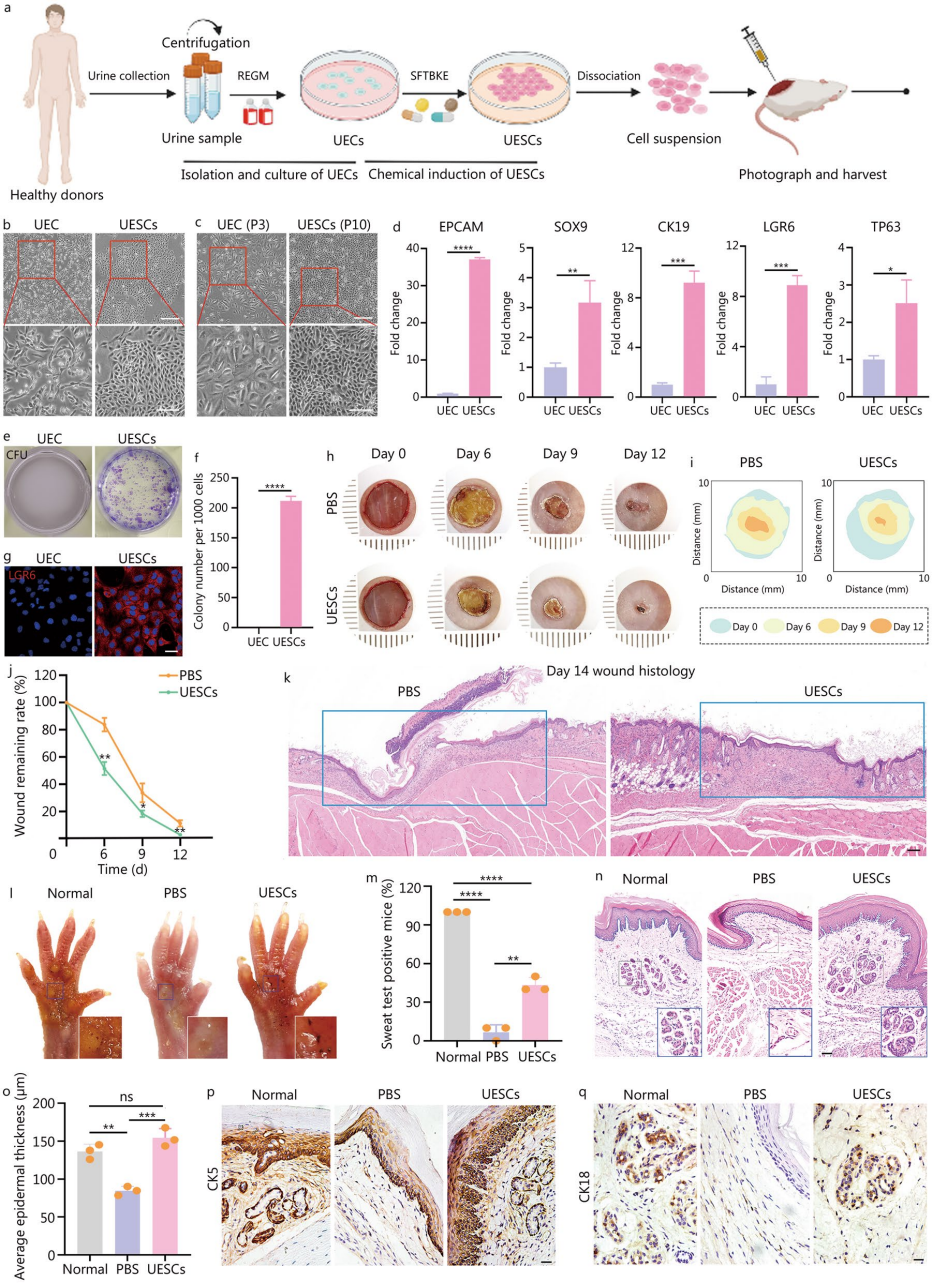
The pharmacological conversion of UECs into UESCs. **a** The scheme of reprogramming of UECs into UESCs by a chemical cocktail. UECs were isolated, and cultured in REGM medium for 2 d, then the medium was changed into inducing medium containing SFTBKE chemical cocktail for 7 d. The UESCs with enough number and quality were in vivo transplanted for skin wound healing and SG restoration. It was created with BioRender.com. **b** Representative morphologies of UECs and UECs-derived UESCs induced by the SFTBKE chemical cocktail. Scale bars = 200 µm. Local magnification: Scale bars = 100 µm. **c** Representative morphologies of third-generation UECs and 10th-generation UESCs. Scale bars = 200 µm. Local magnification: Scale bars = 100 µm. **d** Relative mRNA expression of stem cell-associated markers in UECs and UESCs. **e** Colony formation assay showing stemness and proliferation of UESCs. **f** Quantitative analysis of cloning formation between UECs and UESCs (*n* = 3). **g** Representative immunofluorescence of LGR6 in UECs and UESCs. Scale bar = 50 μm. **h** Representative macroscopic illustration of wound healing at indicated days in PBS and UESCs-treated mice. **i** The wound trace of PBS group and UESCs-treated groups. **j** Quantification of individual wound areas at days 6, 9 and 12, in PBS and UESCs-treated groups in relation to their respective initial size at day 0 (*n* = 10 per group). **k** Hematoxylin and eosin (H&E) stained sections of PBS and UESCs-treated wounds at day 14. Scale bar = 125 μm. **l**, **m** Starch-iodine sweat tests on the paw skin of thermal-injured mice showing only paws of the UESCs-treated mice responded to the assay by displaying indigo-black dots at day 21 (*n* = 10 per independent experiment). **n** H&E staining showing the PBS, UESCs-treated paw skin wounds at day 21 post-injury. Emerging glandular structures were seen in the dermis of UESCs-treated mice. The blue line represents the ridges of the epidermis where the sweat pores open. Scale bars = 50 μm.** o** The average epidermal thickness of normal, PBS and UESCs groups. **p**, **q** The SG markers CK5 and CK18 were assessed by immunohistochemical analysis to examine the SG formation. Scale bars = 50 μm. ns non-significant, ^*^P < 0.05, ^**^P < 0.01, ^***^P < 0.001, ^****^P < 0.0001. Data are presented as the mean ± SE. UECs urine epithelial cells, UESCs urine epithelial stem cells, REGM renal epithelial cell growth medium, EPCAM epithelial cell adhesion molecule, SOX9 SRY-box transcription factor 9, CK cytokeratin, LGR6 leucinerich repeat containing G protein-coupled receptor 6, TP63 tumor protein 63, CFU colony-forming units, PBS phosphate buffered saline, SG sweat glands, mRNA messenger RNA, SE standard error, SFTBKE SB431542, Forskolin, TTNPB, bone morphogenetic protein 4, keratinocyte growth factor, epidermal growth factor

To assess the tissue reconstitution potential of UECs-derived UESCs, 8 mm diameter full-thickness excisional wounds were created on the back of mice (8-week-old female, *n* = 10 in each group). UESCs or phosphate buffered saline (PBS) control was injected intradermally at the wound edge, and the tissues around the wounds were collected for further characterization. The healing process at 0, 6, 9, and 12 d after injection was shown in Fig. 1h. The UESCs-treatment group showed more rapid recovery of wounds as early as day 6. At post-wounding day 12, the wound of the UESCs-treatment group almost completely healed, while there were still large wound areas in the control group. For visualization, the dynamic healing process of each group at different times was traced in the schematic diagram in Fig. 1i. The quantitative results also showed that the wound remaining rate of the UESCs-treated group was smaller than that of PBS group (Fig. 1j). The results of hematoxylin and eosin (H&E) staining in post-wounding skin specimens at day 14 revealed that the cell density and epithelialization degree of granulation tissue in the UESCs group exhibited significant improvement when compared with PBS group (Fig. 1k). These results revealed UESCs can promote rapid re-epithelialization and accelerate cutaneous wound healing.

To evaluate the effect of UESCs on SG restoration, a model of second-degree scald burns in the mouse footpads was created (*n* = 10 in each independent experiment). Post-transplantation for 21 d, the burned footpads in all the groups have recovered. Starch-iodine sweat test analysis revealed that approximately (43.3 ± 5.77)% of UESCs-treated mice showed indigo-black dots, which was significantly higher than those of PBS group (6.67 ± 5.77)% (Fig. 1l, m). The H&E staining analysis result indicated that, similar to the normal group, plantar skin in UESCs group restored rete ridges, which was essential for weight-bearing function, while PBS group did not. Besides, intact SG with evident ductal and glandular structures were also observed in the dermis of UESCs-treated mice (Fig. 1n). Of note, the re-epithelialized wound in footpads of the UESCs-treated group had a thicker epidermis than that of PBS group (Fig. 1o). We additionally observed there was the similar expression of the SG markers CK5 and CK18 in UESCs-treated group and the normal group, while no SG was observed in PBS group (Fig. 1p, q). Here, the mechanisms by which the UESCs restore SG formation may include: 1) activating endogenous SG stem cells via paracrine effect; 2) inducing in situ transdifferentiation of epidermal stem cells into SG cells; 3) the differentiation of UESCs into SG cells. Human-specific antibody CK18 was used to test the potential mechanisms via immunohistochemistry assay, and the results showed that UESCs may have the potential to differentiate into SG cells in vivo (Additional file 1: Fig. S2).

In this study, we successfully achieved chemical induction of human UECs into UESCs to repair skin wounds functionally, which may be an attractive strategy for clinical application and remains to be revealed in the future.

### Supplementary Information

**Additional file 1**. Material and methods. **Table S1** Chemicals and recombinant proteins used in the study. **Table S2** Primer sequences used in the study.  **Fig. S1** The viability and number of UECs directly obtained from human samples. **Fig. S2** Human-specific antibody CK18 were assessed by immunohistochemical analysis to examine the potential transdifferentiation of UESCs into SG in vivo.

## Data Availability

The data and materials used in the current study are all available from the corresponding author upon reasonable request.

## Abbreviations

CK: Cytokeratin
H&E: Hematoxylin and eosin
LGR6: Leucinerich repeat containing G protein-coupled receptor 6
PBS: Phosphate buffered saline
SFTBKE: SB431542, Forskolin, TTNPB, bone morphogenetic protein 4, keratinocyte growth factor, epidermal growth factor
SG: Sweat glands
UECs: Urinary epithelial cells
UESCs: Urinary epithelial stem cells
