# Supplementary material for: Human urinary cells for functional wound healing with sweat gland restoration
Source: Mil Med Res. 2023 Nov 28;10:57. doi: 10.1186/s40779-023-00492-6 (PMC10683131; doi:10.1186/s40779-023-00492-6)
Supplement: Supplementary file 1 — Additional file 1. Material and methods. Table S1 Chemicals and recombinant proteins used in the study. Table S2 Primer sequences used in the study. Fig. S1 The viability and number of UECs directly obtained from human samples. Fig. S2 Human-specific antibody CK18 were assessed by immunohistochemical analysis to examine the potential transdifferentiation of UESCs into SG in vivo. [file 40779_2023_492_MOESM1_ESM.pdf]

Material and methods

Isolation and culture of human urinary epithelial cells

Urinary epithelial cells (UECs) were isolated from healthy male adult donors using methods described previously [1]. In detail, UECs were isolated from urine samples from 10 healthy males aged 25 – 30 years old. Briefly, a total of 10 fresh voided urine samples (200 ml each person) supplemented with 1% penicillin-streptomycin solution were collected, and centrifuged at 400 × *g* for 10 min. The cell pellet with 5 ml of supernatant was resuspended in 10 ml of PBS and centrifuged again at 200 × *g* for 10 min. After the final centrifugation and the aspiration of supernatant, the precipitated cells were resuspended in 2 ml of complete medium containing Dulbecco's Modified Eagle Medium/Nutrient Mixture F-12 (DMEM/F12, Gibco, USA), 10% FBS (Gibco, USA), 1% penicillin-streptomycin solution, and several supplements [10 ng/ml epidermal growth factor (EGF), 1 μmol/L epinephrine, 0.5 μg/ml hydrocortisone, 5 μg/ml transferrin, 5 ng/ml bovine insulin, 0.18 mmol/L adenine, and 1.37 nmol/L 3,3,5-triiodo-L-thyromine)] and seeded into a single well of a 12-well plate (coated beforehand with 20 μg/ml collagen IV). The cells were incubated in a humidified atmosphere with 5% CO_2 at 37 °C. When UECs emerged, the culture medium was replaced with proliferation medium including renal epithelial cell growth medium (REGM) supplemented with Renal Growth BulletKit (CC-3191 & CC-4127, Lonza, USA). REGM was refreshed every other 3 d, and UECs were passaged until they reached approximately 80% confluence.

Quantitative real-time polymerase chain reaction

Cell total RNA was extracted with TRIzol reagent (Invitrogen, 15596-018), as recommended by the manufacturer. PrimeScript RT reagent kit (TaKaRa, Japan) was used to reverse the transcription of cDNA. RT-PCR was performed, and SYBR Green Supermix (Bio-Rad, USA) was used for relative quantification of the indicated genes. Relative gene expression was analyzed based on the $2^{-\Delta\Delta Ct}$

method with β-actin as internal control. The primer sequences for RT-PCR are listed in Table S2.

Colony formation assay

For colony formation assay, 1000 cells/well were plated in a 6-well plate and incubated for 10 d. Cells were fixed in 4% paraformaldehyde (Solarbio, China) at room temperature for 10 min, and then stained with 0.1% crystal violet for 30 min. The photographs were taken by an EOS digital SLR.

Immunofluorescence and immunohistochemistry

For immunofluorescence staining, cells were fixed in 4% paraformaldehyde at room temperature for 30 min and permeabilized in 0.2% Triton ×100 in PBS (PBST) for 10 min. The cells were then blocked with PBST containing 5% normal goat serum at room temperature for 30 min. Then cells were probed with primary antibodies at 4 °C overnight and secondary antibodies at room temperature for 2 h. The primary antibodies and secondary antibodies used in this study were rabbit anti-LGR6 (1:200, ab240030, Abcam, USA) and goat anti-rabbit IgG H&L (Alexa Fluor 594) (1:200, ab150080, Abcam, USA).

For immunohistochemistry staining, antigen retrieval of samples was performed in 10 mmol/L citric acid buffer (pH = 6.0) for 15 min, and 0.3% H_2O_2 was added to block endogenous peroxidase activity. Ten slides were incubated with rabbit anti-CK5 (1:200, ab52635, Abcam, USA) and rabbit anti-CK18 (1:200, ab133263, Abcam, USA) overnight at 4 °C. To test the potential transdifferentiation of UESCs into SG in vivo, human-specific monoclonal antibody CK18 (1:200, ab7797, Abcam, USA) was used. Antibody binding was detected with a streptavidin–biotin-peroxidase immunohistochemical system (SP-9000, ZSGB-BIO), and color development was detected through DAB staining (ZLI-9017, ZSGB-BIO). The slides were counterstained with hematoxylin.

Animals and transplantation

The animal experiment was performed according to protocols Institutional Animal Care and Use

Committee (IACUC) guidelines, and it has been approved by the Ethics Committee at the Fourth Medical Center of PLA General Hospital. For plot full-thickness excisional wound-healing assays, 8 mm diameter full-thickness excisional wounds were created on the back of mice. Eight-week-old female athymic BALB/c nude mice were used and randomly divided into a control group with PBS injections (n = 10) and UESCs transplantation group (n = 10), in which the UESC number is 1×10^6 used via multipoint and multiday wound edge injection. After the injection, mice were monitored daily and sacrificed at indicated days. Wound remaining rate (%) = actual wound area/initial wound area × 100%. For sweat gland regeneration assays, 8-week-old female athymic BALB/c nude mice were used for establishing a mouse burn model. Mice were anesthetized with pentobarbital and a second-degree scald wound was created on the hind paws of mice using an electrical scald instrument for 3 s to partially destroy subdermal sweat glands [2]. Then, UESCs (5×10^5 cells in 50 µl medium) were intradermally injected into the paw pads of recipient mice (n = 10 in each independent experiment). Simultaneously, 50 µl of PBS was injected into the scalded paw pads as vehicle controls (n = 10 in each independent experiment). Three independent experiments were conducted in each group, respectively.

Sweat tests

The paw pads were smeared with 2% (w/v) iodine/ethanol solution evenly, followed by starch. After drying, 25 µl acetylcholine (100 µmol/L, Sigma-Aldrich) was injected subcutaneously into the paws of the mice. The sweat test was performed weekly in the first 2 weeks and every 3 d in the next weeks to evaluate the function of nascent sweat gland.

Statistics

Statistical analysis was performed with GraphPad Prism 8.0 software. All data shown in the graphs are presented as means ± standard error (SE). The data follows a normal distribution and the variances

were homogeneous. Comparisons between two groups were analyzed by Student' *t*-test, and one-way or two-way ANOVA was used for more than two groups. The value of $P < 0.05$ was considered statistically significant and marked with an asterisk. ns represented non-significant; $^{*}P < 0.05$; $^{**}P < 0.01$; $^{***}P < 0.001$; $^{****}P < 0.0001$.

Table S1 Chemicals and recombinant proteins used in the study

Chemicals	Mechanism of action	Concentration	Source	Cat#
SB431542	TGF-β inhibition	10 μmol/L	Selleck	S1067
Forskolin	cAMP agonist	10 μmol/L	Selleck	S2449
TTNPB	RAR agonist	1 μmol/L	Selleck	S4627
BMP4	Smad1/5/8	20 ng/ml	R&D	314-BPE
KGF	FGFR2 activation	20 ng/ml	Preprotech	100-19
EGF	ERK/MAPK	20 ng/ml	Sigma	E5036

BMP4 bone morphogenetic protein 4, *KGF* keratinocyte growth factor, *EGF* epidermal growth factor, *FGFR2* fibroblast growth factor receptor 2, *ERK/MAPK* extracellular signal-regulated kinases/ mitogen-activated protein kinases, *RAR* retinoic acid receptors, *cAMP* adenosine 3',5'-cyclic monophosphate, *TGF-β* transforming growth factor-β

Table S2 Primer sequences used in the study

Primers	Sequences (5' to 3')
SOX9	Forward: AGACCTTTGGGCTGCCTTAT
	Reverse: TAGCCTCCCTCACTCCAAGA
LGR6	Forward: AGCCCTGTGAGTACCTCTTTG
	Reverse: CCACAGGAAATGCCAGTCAA
EPCAM	Forward: ATAACCTGCTCTGAGCGAGTG
	Reverse: TGCAGTCCGCAAACTTTTACTA
CK19	Forward: TGAGTGACATGCGAAGCCAAT
	Reverse: CTCCCGGTTCAATTCTTCAGTC
TP63	Forward: GGACCAGCAGATTCAGAACGG
	Reverse: AGGACACGTCGAAACTGTGC
β-actin	Forward: CATGTACGTTGCTATCCAGGC
	Reverse: CTCCTTAATGTCACGCACGAT

SOX9 SRY-box transcription factor 9, *LGR6* leucine-rich repeat-containing G-protein-coupled receptor 6, *EPCAM* epithelial cell adhesion molecule, *CK19* cytokeratin 19, *TP63* tumor protein 63

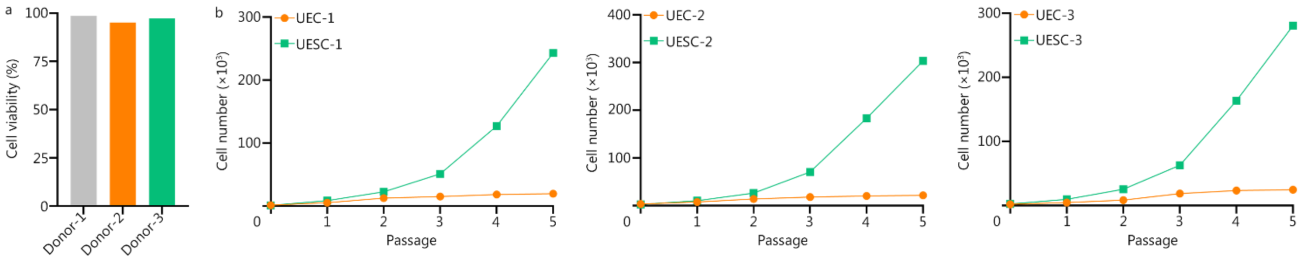

Fig. S1 The viability and number of UECs directly obtained from human samples. **a** The cell viability of the UECs at passage 0. **b** The quantitative measurement of cell number at passage 0 – 5 for 3 donors. UEC urinary epithelial cell, UESC urinary epithelial stem cell

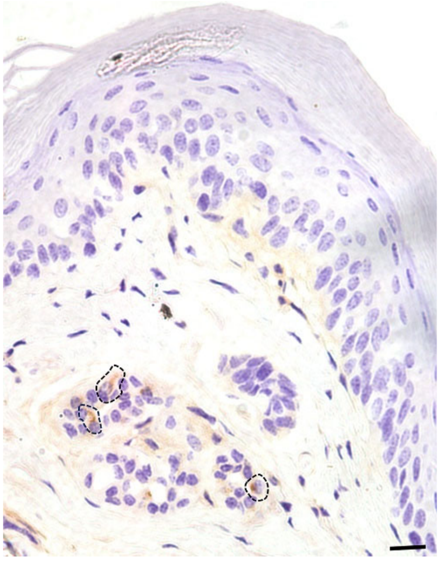

Fig. S2 Human-specific antibody CK18 were assessed by immunohistochemical analysis to examine the potential transdifferentiation of UESCs into SG in vivo. The black dotted line represents human-specific CK18-postive staining. Scale bar = 50 µm.

References

1. Zhou T, Benda C, Dunzinger S, Huang Y, Ho JC, Yang J, et al. Generation of human induced pluripotent stem cells from urine samples. Nat Protoc. 2012;7(12):2080-9.
2. Chen H, Liu Y, Liu Y, Ji S, Xiang J, Li Y, et al. Biomimetic small exosome with outstanding surgical applications for rapid large-scale wound healing and functional sweat gland restoration. Nano Today. 2022;45:101531.
